# Direct Synthesis of Single-Crystalline Bilayer Graphene on Dielectric Substrate

**DOI:** 10.3390/nano15211629

**Published:** 2025-10-25

**Authors:** Zuoquan Tan, Xianqin Xing, Yimei Fang, Le Huang, Shunqing Wu, Zhiyong Zhang, Le Wang, Xiangping Chen, Shanshan Chen

**Affiliations:** 1 School of Physics and Beijing Key Laboratory of Optoelectronic Functional Materials and Micro-Nano Devices, Renmin University of China, Beijing 100872, China; tzq@ruc.edu.cn (Z.T.); 17853530505@163.com (X.X.); le.wang@ruc.edu.cn (L.W.); 2 Key Laboratory of Quantum State Construction and Manipulation (Ministry of Education), Renmin University of China, Beijing 100872, China; 3 School of Science, Jimei University, Xiamen 361021, China; ymfang@jmu.edu.cn; 4 Key Laboratory for the Physics and Chemistry of Nanodevices and Center for Carbon-Based Electronics, School of Electronics, Peking University, Beijing 100871, China; huanglenku@126.com (L.H.); zyzhang@pku.edu.cn (Z.Z.); 5 Collaborative Innovation Center for Optoelectronic Semiconductors and Efficient Devices, Department of Physics, Xiamen University, Xiamen 361005, China; 6 Tsinghua Shenzhen International Graduate School, Tsinghua University, Shenzhen 518055, China

**Keywords:** Bernal-stacked bilayer graphene, single-crystalline, remote catalysis, homoepitaxial growth, dielectric substrates

## Abstract

Direct growth of high-quality, Bernal-stacked bilayer graphene (BLG) on dielectric substrates is crucial for electronic and optoelectronic devices, yet it remains hindered by poor film quality, uncontrollable thickness, and high-density grain boundaries. In this work, a facile, catalyst-assisted method to grow high-quality, single-crystalline BLG directly on dielectric substrates (SiO_2_/Si, sapphire, and quartz) was demonstrated. A single-crystal monolayer graphene template was first employed as a seed layer to facilitate the homoepitaxial synthesis of single-crystalline BLG directly on insulating substrates. Nanostructure Cu powders were used as the remote catalysis to provide long-lasting catalytic activity during the graphene growth. Transmission electron microscopy confirms the single-crystalline nature of the resulting BLG domains, which validates the superiority of the homoepitaxial growth technique. Raman spectroscopy and electrical measurement results indicate that the quality of the as-grown BLG is comparable to that on metal substrate surfaces. Field-effect transistors fabricated directly on the as-grown BLG/SiO_2_/Si showed a room temperature carrier mobility as high as 2297 ± 3 cm^2^ V^−1^ s^−1^, which is comparable to BLG grown on Cu and much higher than that reported on in-sulators.

## 1. Introduction

Bernal-stacked bilayer graphene (BLG) features appealing physical properties, including a tunable electronic bandgap [1,2,3] and intrinsic valley degree of freedom [4,5,6], making it a candidate of interest for electronic, optoelectronic, and quantum devices [7,8]. Conventional chemical vapor deposition (CVD) on metallic catalysts—including Cu [9,10,11], Ni [12], and Cu-Ni [13,14,15] alloys—has yielded large-area, high-quality Bernal-stacked graphene (the bilayer grain size ranges between 5 and 40 μm). In contrast, insulating substrates offer limited catalytic activity for precursor dissociation and impose high surface-diffusion barriers for carbon species (in the order of several eV) [16]. A dual-mechanism model has been proposed for insulating substrates in which single-particle deposition and agglomerate-driven formation coexist, demonstrating that an agglomerate-dominated pathway can yield a significantly smoother film [17]. Consequently, direct growth on insulators typically relies on elevated temperatures [18] and prolonged growth times [19], often resulting in non-uniform thickness, high defect densities, and abundant grain boundaries (the monolayer grain size typically ranges from hundreds of nanometers to several micrometers) [19,20], which constrain film quality and limit device applicability [21].

Substantial efforts have been made to achieve the direct growth of graphene on dielectric substrates, including, but not limited to, SiO_2_/Si [22,23,24,25,26,27,28], Si [29,30], Si_3_N_4_ [19,31], sapphire [32,33,34], quartz [35,36,37,38], glass [39], and h-BN [40,41]. These efforts have generally followed two complementary directions: The first is energy regulation, achieved via tailored thermal fields [23,34], plasma assistance [30,32,41], or free molecular flow conditions [37] to enhance precursor activation and favor edge-limited lateral propagation. However, graphene grown via this approach typically exhibits small grain sizes, compromised crystallinity (as evidenced by prominent D band peaks in Raman spectra [42,43]), and uncontrolled layer thickness. The second strategy involves catalytic assistance, such as predeposited thin metal films [22,27,31,33,35], metal vapor-assisted growth [24,25,26,29,36], or non-metallic catalysts (e.g., CO_2_) [28,38,39,40], to facilitate nucleation and lateral expansion. While an interfacial metal catalyst can markedly improve graphene quality, it introduces long-standing issues related to metal removal and metallic residue contamination [33]. Remote metal vapor delivery and non-metallic catalysts can suppress dopant incorporation and avoid post-growth etching [26,29], yet neither strategy overcomes the inherently poor nucleation on bare insulating surfaces, and the resulting films remain highly defective. Moreover, precise control over the number of graphene layers on insulating substrates remains an ongoing challenge.

Here, we report a substrate-agnostic homoepitaxial strategy for the direct synthesis of single-crystalline BLG on dielectric substrates. By employing single-crystalline monolayer graphene (SLG) as a template layer on SiO_2_/Si, sapphire, and quartz, we precisely control the in-plane crystallographic registry. Copper nanopowders (Cu NPs) enable remote activation and flux regulation of CH_4_ decomposition, allowing for fine-tuned supply of reactive carbon species. Through optimization of the temperature–pressure–gas composition window, the process favors second-layer nucleation and lateral growth, yielding BLG with orientation-aligned crystalline inheritance and deterministic layer number control. Comprehensive characterization via Raman spectroscopy and transmission electron microscopy (TEM) confirms a Bernal-stacked structure with exceptional quality, evidenced by negligible D band intensity and sharp G-peak profiles. Field-effect transistors (FET) fabricated directly on graphene/SiO_2_/Si substrate had a carrier mobility as high as 2297 ± 3 cm^2^ V^−1^ s^−1^, which is comparable to that of BLG grown on Cu and much higher than that reported on insulators [29,44].

## 2. Experimental Methods

### 2.1. Preparation of Single-Crystalline SLG Template

Large-area single-crystal graphene was grown on electropolished Cu foils (25 μm thick, Alfa Aesar, Ward Hill, MA, USA, stock No. 46365) using a horizontal quartz tube furnace (Mini-Mite, Lindberg/Blue M, Asheville, NC, USA; quartz tube inner diameter: 1 inch) [45,46,47]. For typical growth, Cu enclosure underwent vacuum annealing at 1030 °C for 10 min (partial pressure: 2 mTorr; heating rate: ~10 °C/min), followed by graphene deposition in 1 sccm CH_4_ and 50 sccm H_2_ for 3 h (partial pressure: 586 mTorr). After growth, the system was cooled down rapidly to room temperature under the same gaseous atmosphere (cooling rate: ~15 °C/min). The resulting graphene films on the inner surface of Cu enclosure were transferred onto various dielectric substrates (e.g., 300 nm SiO_2_/Si, sapphire and quartz) using the PMMA-assisted wet transfer method [45]. After wet transfer, monolayer graphene on dielectric substrates shows minor damage and PMMA residue. The residual polymer can be removed through subsequent annealing, while the limited broken area does not significantly affect the growth of the BLG.

### 2.2. Synthesis of Single-Crystalline BLG

The synthesis of single-crystal BLG on single-crystal SLG was carried out in the same quartz tube furnace (Mini-Mite, Lindberg/Blue M, Asheville, NC, USA; quartz tube inner diameter: 1 inch) under a low-pressure CVD system. Prior to the growth process, the quartz tube was evacuated to below 0.5 Pa using a mechanical pump to remove residual gases. Cu NPs (10–30 nm, Aladdin, Shanghai, China, stock No. K1419065) were employed as the floating catalysts, strategically positioned in the upper gas stream to enable remote activation of hydrocarbon decomposition. The insulating substrates (size: 25 mm × 20 mm) with pre-transferred SLG were loaded into the center of the quartz tube furnace. The system was heated up to 1000 °C at a rate of ~10 °C/min and then thermally annealed for 2 h under 10 sccm H_2_ (partial pressure: 120 mTorr) to remove transfer-induced residues and achieve pristine graphene seeding surfaces. CH_4_ flow rates were systematically varied (5, 10, 15, and 20 sccm) to modulate carbon flux. Following 1 h exposure to the CH_4_/H_2_ mixture, the system underwent rapid quenching to room temperature to terminate growth.

### 2.3. Characterizations

Scanning electron microscopy (SEM, Zeiss Sigma, Carl Zeiss AG, Oberkochen, Germany) was utilized to investigate the surface morphology of the as-grown graphene samples. The imaging conditions were maintained at 10 kV accelerating voltage with a 9.0 mm working distance using an InLens secondary electron detector. Transmission electron microscopy (TEM, FEI TECNAI G^2^ F20, Thermo Fisher Scientific, Hillsboro, OR, USA, operated at 200 kV) was used to analyze the crystallinity and stacking order of bilayer graphene. Raman spectroscopy (Alpha 300, WITec GmbH, Ulm, Germany) with a 488 nm laser was performed at ambient temperature to evaluate the structural quality, layer number, and stacking configuration of the as-grown graphene.

### 2.4. Device Fabrication and Electrical Measurements

The electrical properties of the single-crystal BLG were characterized by fabricating dual-gate graphene FET devices directly on the as-grown graphene on 300 nm SiO_2_/Si substrates. The device fabrication was based on electron-beam lithography (EBL, Raith eLINE Plus system, Raith GmbH, Dortmund, Germany). A polymethyl methacrylate (PMMA, 950 K A4, MicroChem Corp., Newton, MA, USA) resist layer with a thickness of ~200 nm was spin-coated and patterned to define the graphene channel with dimensions of L/W = 9 μm/5 μm, followed by etching of excess graphene using oxygen plasma (100 W, 40 s). Subsequently, a 3 nm thick yttrium strip was deposited across the channel by e-beam evaporation (Kurt J. Lesker PVD 75, Kurt J. Lesker Company, Jefferson Hills, PA, USA). After exposure in air at 240 °C for 30 min, yttrium oxide (Y_2_O_3_, ~5 nm) was formed above the strip. This Y_2_O_3_ buffer layer acts as a seed layer to improve the nucleation and interface quality for the subsequent high-κ dielectric. A 20 nm HfO_2_ layer was then deposited by atomic layer deposition (ALD, Beneq TFS 200, Beneq Oy, Espoo, Finland), serving as the top gate dielectric in conjunction with the Y_2_O_3_ layer. Finally, Ti/Au (5/30 nm) source, drain, and gate electrodes were deposited by e-beam evaporation. The Ti layer acts as an adhesion layer to the underlying graphene, while Au ensures low contact resistance. The doped silicon substrate functioned as the back gate. Electrical measurements were performed in air at room temperature using a Keithley 4200SC semiconductor parameter analyzer (Tektronix Inc., Beaverton, OR, USA).

## 3. Results and Discussion

**Figure 1:**
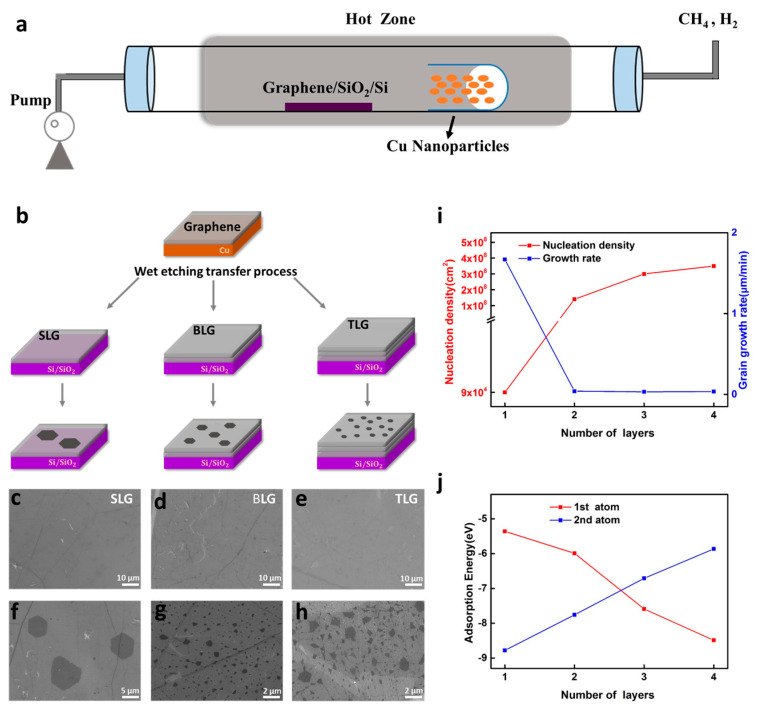
Cu NP-assisted, graphene seeding layer-dependent homoepitaxy of graphene on dielectric substrates. (**a**) Schematic of the low-pressure CVD system employing floating Cu NPs under CH_4_/H_2_. (**b**) Workflow: SLG growth on Cu, wet transfer onto dielectrics, followed by homoepitaxial overgrowth. (**c**–**e**) SEM images of the as-transferred SLG, BLG, and TLG seeding layers on 300 nm SiO_2_/Si, acquired prior to the epitaxial growth step. (**f**–**h**) SEM images after 1 h of homoepitaxial growth, showing quasi-hexagonal adlayer graphene domains on the corresponding graphene template in (**c**–**e**). (**i**) Nucleation density (left axis) and lateral grain growth rate (right axis) as functions of seeding layer thickness (number of graphene layers). (**j**) DFT-calculated adsorption energies for the first and second C atoms versus seeding layer thickness.

Figure 1a schematically depicts the Cu NP-assisted, SLG-template-based BLG growth technique. A small quartz tube sealed at one end was loaded with Cu NPs and positioned with its open end facing downstream, ~8 cm from the dielectric substrate. This distance, optimized to balance catalytic efficacy against particle contamination, furnishes the maximum enhancement of growth without depositing Cu nanocrystals on the wafer. Cu NPs were employed as a remote catalyst due to their nanoscale morphology and high specific surface area, which provide a stronger and more sustained catalytic activity than Cu foil [44,47]. The Cu nanoparticles exist in a granular form, with an average grain size of 23.7 ± 7.9 nm (see Figure S1 in Supplementary Materials for more details). The remote catalytic effect of Cu NPs plays a crucial role in enabling the growth of bilayer graphene (BLG). Our findings demonstrated that without these Cu NPs, graphene growth cannot be achieved on graphene–SiO_2_/Si platforms (see Figure S2 in Supplementary Materials for more details). SEM and Raman characterizations revealed that while graphene signals were detected on certain areas of the Cu NPs after 1 h and 3 h growth, most regions remained uncovered, indicating that the majority of the Cu NPs retained their catalytic activity (see Figure S3 in Supplementary Materials for more details).

To further clarify how the graphene template influences homoepitaxial adlayer formation, we compared growth on monolayer, bilayer, and trilayer graphene templates (Figure 1b). After 1 h of growth, the secondary graphene adlayer domains had nucleated on all three templates (Figure 1c–h). It is noticed that the domain size and nucleation density of the as-grown graphene varies with the thickness of the seeding layer. As shown in Figure 1i, the nucleation density increases as the number of graphene seeding layers rises from one to three, whereas the lateral grain growth rate drops sharply from one to two layers and then plateaus. To gain a thorough understanding of the evolution of the growth behavior, density functional theory (DFT) calculations were carried out (see Section S2 in Supplementary Materials for more details). With an increasing number of graphene seeding layers (from one to four), the adsorption energy of the first C atom decreases progressively from −5.89 eV to −8.78 eV, while that of the second C atom increases markedly from −8.49 eV to −5.35 eV (Figure 1j). These results indicate that, as the seeding layer becomes thicker, lateral enlargement of existing adlayer islands requires more energy than the formation of new nuclei, which accounts for the experimentally observed high-nucleation/low-growth regime (Figure 1f–h). Furthermore, no graphene nuclei form on bare SiO_2_/Si after 1 h growth. Extending the growth time to 3 h yields only low-quality micron-sized flakes (see Figure S5 in Supplementary Materials for more details). Taken together with the DFT results, these observations underscore the crucial role of the graphene seeding layer in promoting and directing the homoepitaxial growth of adlayer graphene. Specifically, a monolayer graphene template delivers an optimal nucleation density for the adlayer while imposing only a modest energy barrier to lateral island expansion, thereby enabling reproducible growth of bilayer graphene. Moreover, because the energy barrier for lateral expansion after nucleation on monolayer graphene is lower than that for secondary nucleation on an existing bilayer, only a second graphene layer forms on the monolayer seed; further multilayer growth is suppressed. This intrinsic selectivity enables uniform, layer-precise synthesis of strictly bilayer graphene.

**Figure 2:**
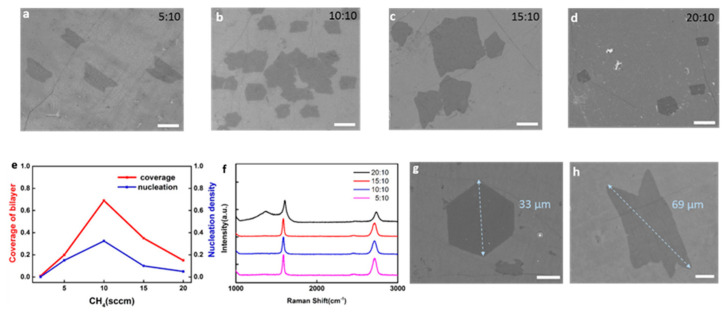
Effect of CH_4_ supply on BLG growth on SLG/SiO_2_/Si substrates. (**a**–**d**) SEM images of BLG synthesized on SLG/SiO_2_/Si substrates under identical conditions except for the CH_4_/H_2_ ratio for 5:10 (**a**), 10:10 (**b**), 15:10 (**c**), and 20:10 (**d**). (**e**) Areal coverage and nucleation density of BLG as functions of CH_4_ flow at a fixed H_2_ flow of 10 sccm. (**f**) Representative Raman spectra of samples grown at various CH_4_ flows. (**g**,**h**) SEM images of BLG domains with hexagonal and lobed morphologies; the largest diagonal reaches 69 μm. Scale bar: 10 μm.

The key variables for Cu NP-assisted graphene seeding growth are the annealing duration and CH_4_ concentration. In contrast to the typical process for graphene growth, we annealed the substrates at 1000 °C in H_2_ flow for 2 h to remove transfer residues and activate growth sites. Typically, no growth would happen during the first 2 h, even with CH_4_ flow throughout the whole procedure. In addition, the effect of CH_4_ concentration was studied by varying CH_4_ flow while maintaining H_2_ flow at the same level (10 sccm). Figure 2a–d show the SEM images of BLG with different growth gas ratios (including CH_4_:H_2_ = 5:10, 10:10, 15:10, and 20:10). Both the areal coverage and the nucleation density first increase and then decrease with the increasing CH_4_ flow, yielding an inverted V dependence (Figure 2e). Some BLG domains even merge together to form the continuous film in Figure 2b. Raman spectra acquired from samples grown at the different CH_4_ flows (Figure 2f) indicate high-quality BLG (negligible D band) for CH_4_ ≤ 15 sccm, whereas at 20 sccm, a pronounced D band and a broadened G band emerge, consistent with reduced crystallinity [43,48]. As a consequence, the condition of CH_4_:H_2_ = 10:10 can be considered as the optimized condition to obtain BLG with higher growth efficiency. Under the optimized condition (CH_4_:H_2_ = 10:10), large BLG domains with hexagonal or lobed morphologies are obtained, with diagonal lengths up to 69 µm after 1 h on SLG/SiO_2_/Si (Figure 2g,h)—substantially larger than the ~11 µm monolayer domains that were directly synthesized on SiO_2_/Si after 72 h [19]. Furthermore, this Cu NP-assisted seeding approach is also applicable to other dielectric substrates, such as quartz and sapphire (see Figure S6 in Supplementary Materials for more details).

**Figure 3:**
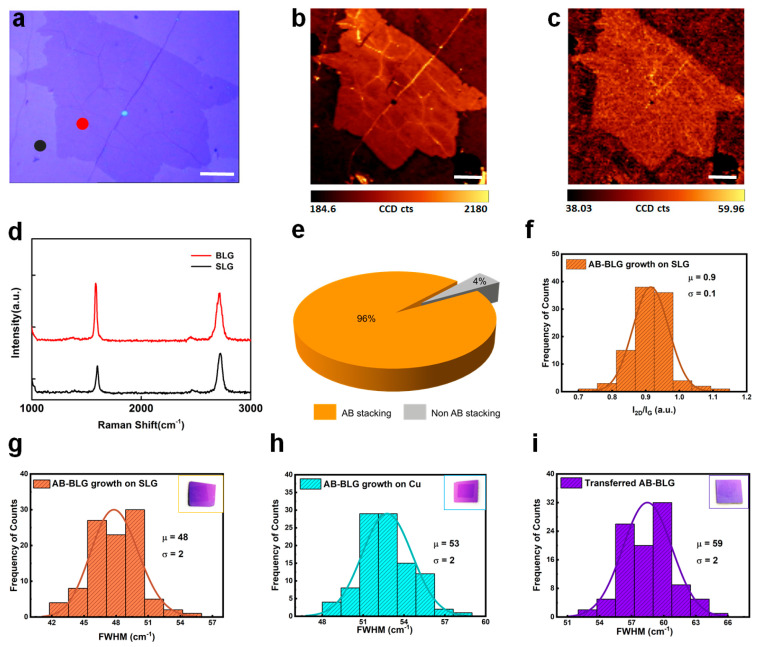
Raman measurements of the as-grown BLG. (**a**) Optical image of a BLG domain with the colored dots indicating Raman measurement positions corresponding to the spectra in (**d**). (**b**,**c**) Raman map of G band intensity (1520–1640 cm^−1^) and 2D band FWHM (2640–2780 cm^−1^) of the same BLG grain as in (**a**). Scale bar in (**a**–**c**): 10 μm. (**d**) Raman spectra of the SLG and BLG areas marked in (**a**). (**e**) Stacking ratio of the BLG grown based on optimized condition. (**f**) Histogram of the I_2D_ /I_G_ ratio for the BLG grown through the seeding technique. (**g**–**i**) Histograms of the Raman spectrum 2D band FWHM values of BLG grown through the seeding technique (**g**), directly grown on Cu foil (**h**), and by transferring additional monolayer graphene onto the SLG/SiO_2_/Si substrate (**i**). The number of bins in each histogram was determined according to Sturges’ criterion (C = 1 + 3.322lgN), with distributions being further fitted using appropriate probability density functions (lognormal) to extract the mean and standard deviation values. Insets: optical images of the corresponding samples transferred onto 300 nm SiO_2_/Si substrates.

The stacking order and uniformity of the as-grown bilayer graphene were further evaluated by Raman spectroscopy. Figure 3a presents an optical micrograph of a representative BLG domain. Raman spectra of the homoepitaxial bilayer (Figure 3d), recorded under the same excitation condition as its monolayer template, reveal a negligible D band, a sharp and intensified G band, and a broadened 2D band, which are characteristic signatures of high-quality AB-stacked BLG [42,43]. Figure 3b,c present the Raman maps of the G band intensity (1520–1640 cm^−1^) and the full width of half maximum (FWHM) of 2D band (2620–2780 cm^−1^) from the region shown in Figure 3a. In the G band map (Figure 3b), the darker contrast denotes SLG regions, whereas the brighter areas correspond to BLG domains. The consistently narrow FWHM values revealed in the 2D band map (Figure 3c) further support that the BLG domains exhibit Bernal stacking. Statistical analysis of the BLG domains obtained under optimized growth conditions shows that AB-stacked bilayer graphene accounts for ~96% of the total bilayer area (Figure 3e).

To provide a statistically rigorous description of the spectral features, the histograms of the I_2D_ /I_G_ ratio and 2D band FWHM are reconstructed in accordance with Sturges’ criterion (C = 1 + 3.322lg(N)), where N is the number of spectra analyzed (approximately 100 in this case, yielding C ≈ 8) [49]. As shown in Figure 3f,g, the distributions were further fitted with lognormal to extract mean values and standard deviations. The I_2D_/I_G_ ratio of the as-grown BLG is 0.9 ± 0.1, while the FWHM of the 2D band is 48 ± 2 cm^−1^. For comparison, the 2D band’s FWHMs obtained from transferred AB-stacked BLG directly synthesized on Cu foil (Figure 3h, 53 ± 2 cm^−1^) and from mechanically stacked bilayers prepared by assembling two monolayer graphene films on SiO_2_/Si (Figure 3i, 59 ± 2 cm^−1^) are significantly larger. These results indicate that BLG grown on the seeding layer exhibits a narrower FWHM, thereby reflecting superior structural uniformity and higher crystalline quality [42,50].

**Figure 4:**
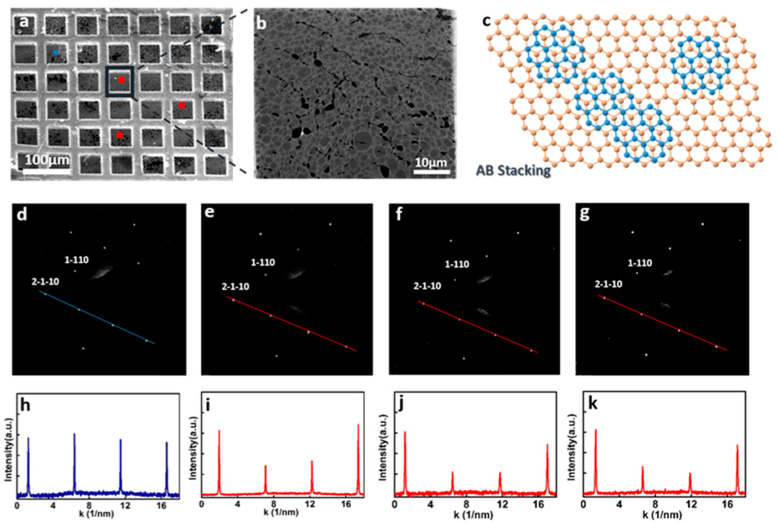
Crystalline structure of the as-grown BLG. (**a**) SEM image of the as-grown BLG transferred onto the TEM grid. The blue and red circles denote the single-crystal graphene template and as-grown BLG area, respectively. (**b**) Higher magnification SEM image of the corresponding black square marked in (**a**). (**c**) A schematic drawing of the synthesis of Bernal-stacked BLG on the single-crystal graphene template. (**d**) SAED pattern of the monolayer graphene template in the blue circle region marked in (**a**). (**e**,**f**) SAED patterns of the as-grown BLG in the red circle regions marked in (**a**); all three regions have the same orientation as the monolayer graphene template. (**h**–**k**) Intensity profiles along the lines in (**d**–**g**), respectively.

The epitaxial growth nature was further evidenced by the crystalline structure of the as-grown BLG, studied by transmission electron microscopy (TEM). The BLG film grown on large single-crystal SLG seeding substrate was further transferred onto the bare copper TEM grid. In the low-magnification SEM image (Figure 4a), blue and red circles mark the SLG seed and the overgrown BLG regions, respectively. Typical selected area electron diffraction (SAED) pattern of the seeding graphene in Figure 4a generates a single set of 6-fold symmetric diffraction spots (Figure 4d), with the diffraction intensity ratio of the outer (2-1-10) peak to the inner (1-110) peak being approximately equal to 1 (Figure 4h), indicating the single-crystalline nature of the monolayer graphene seeding layer. Meanwhile, SAED patterns (Figure 4e–g) from the marked red regions in Figure 4a show similar diffraction patterns as the seeding monolayer graphene, with the diffraction intensity ratio of the outer peak to the inner peak being approximately equal to 2 (Figure 4i–k), which evidenced the AB stacking order of the as-grown bilayer film. Since the investigated samples are only one to two atomic layers thick, the thin-film approximation can be considered valid in this context, allowing for the use of relative diffraction intensities to infer stacking order and crystallinity, as reported in previous studies on few-layer graphene [51,52]. The same crystalline orientation of the bilayer graphene confirms that the second layer of graphene was epitaxially grown on the transferred single-crystal monolayer graphene, as expected [44,53]. As schematically illustrated in Figure 4c, multiple BLG nuclei form with perfect rotational registries on the single-crystal seed and subsequently coalesce into a continuous single-crystal layer without grain boundary defects, even at a high nucleation density.

**Figure 5:**
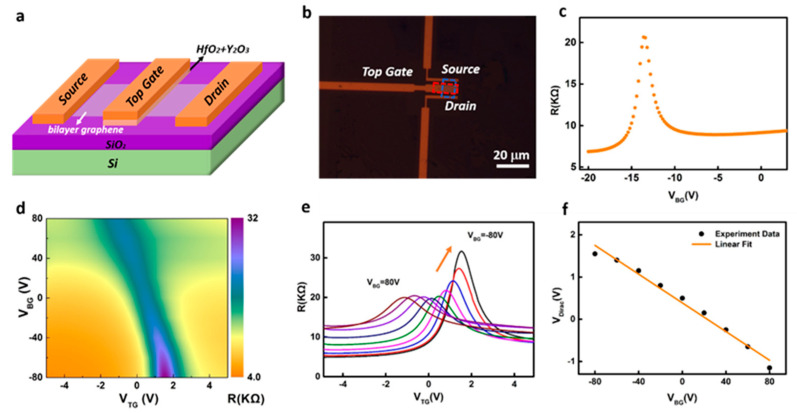
Electrical properties of the as-grown BLG. (**a**,**b**) Schematic structure diagram (**a**) and optical microscope image (**b**) of the BLG device with dual gates applied for the transfer characteristics measurements. The BLG sheet and dielectric layer are marked by the blue and red dashed frame, respectively. (**c**) Plots of device resistance (*R*) versus back gate voltage (*V*_BG_). (**d**) Two-dimensional plot of resistance as functions of both top gate voltage (*V*_TG_) and *V*_BG_ of the dual-gate BLG device. (**e**) Resistance measured as a function of the top gate voltage (*V*_TG_) at a range of fixed back gate voltages (*V*_BG_) from −80 V to + 80 V, the different colors of the lines correspond to different back gate voltages (*V*_BG_). The traces were taken at 20 V steps in the back gate voltage. (**f**) Linear relation between the top gate neutral points and the back gate voltages.

To further evaluate the electronic quality of the BLG films, we fabricated dual-gate graphene field-effect transistors (FETs). Dual-gate architecture was employed to provide stronger electrostatic control of the BLG channel, suppress substrate-induced charge inhomogeneity, and allow for accurate extraction of the intrinsic transport properties of the device [54,55]. The schematic structure of the device is shown in Figure 5a (see Section 2 for more details). Because hafnium oxide (HfO_2_) film could not be directly grown on graphene layers by atomic layer deposition (ALD) [56], an ultrathin yttrium oxide (Y_2_O_3_) film (5 nm) was first deposited, followed by 20 nm HfO_2_ to provide ideal gate dielectric for graphene-based FETs [57,58]. Figure 5b shows the representative optical image of a dual-gate BLG device. The resistance *R* versus the back-gated voltage (*V*_BG_) (*R*-*V*_BG_) plot in Figure 5c shows typical ambipolar characteristics expected for the graphene device. The back gate mobility was extracted to be 2297 ± 3 cm^2^/Vs based on the fitting method reported previously [56]. Furthermore, we swept the voltage of the top gate (*V*_TG_) from −4 V to 4 V while different back gate voltages (*V*_BG_) from −80 V to 80 V were applied on the silicon substrate. The two-dimensional resistance *R* map as a function of *V*_TG_ and *V*_BG_ reveals peak resistance values in regions of the maximum displacement field (top-middle left and bottom-middle right) (Figure 5d). This can be more evidently illustrated in a series of plots of *R*-*V*_TG_ at different *V*_BG_ values in Figure 5e, confirming the AB stacking nature of the bilayer graphene [59]. For each trace, a resistance maximum, corresponding to the charge neutrality point (CNP), was seen. The CNP resistance is adjusted by tuning the vertical electric field, which proves the formation of a tunable band structure [55,60]. This was confirmed by the observation of an enhancement in the current on/off ratio. Figure 5e also shows that the *I*_on_/*I*_off_ values obtained in all measurements were about 1.66 and 6.46 for *V*_BG_ = 80 V and −80 V, respectively. Figure 5f shows that *V*_Dirac_ and *V*_BG_ are linearly related with a slope of about −0.017, which agrees well with the expected value of −*ε*_BG_ *d*_TGall_/*ε*_TGall_ *d*_BG_ = −0.027, where *ε* and *d* correspond to the dielectric constant and thickness of the top gate (Y_2_O_3_: *d*_1_ = 5 nm, *ε*_1_ = 12; HfO_2_: *d*_2_ = 20 nm, ε_2_ = 12, *d*_TGall_ = 25 nm, *ε*_TGall_ = 12) and the bottom gate (SiO_2_: *d*_BG_ = 300 nm, *ε*_BG_ = 3.9). The carrier mobility of the top gate BLG on SiO_2_/Si substrates is 2297 ± 3 cm^2^/Vs, which is comparable to those of CVD-grown bilayer graphene on metal substrates [11]. These results clearly demonstrate that the directly epitaxial grown bilayer graphene on Si wafer is high-quality, and the proposed strategy provides a rational route for synthesizing high-quality AB-stacked bilayer graphene directly on dielectric substrates.

## 4. Conclusions

In conclusion, we have proposed a new method to grow single-crystalline bilayer graphene directly on various dielectric substrates. The quality and stacking order of the bilayer graphene were confirmed by Raman spectroscopy, TEM diffraction patterns, and dual-gated FET measurements. Transport measurements show that the bilayer AB-stacked graphene has a typical tunable transport band gap. The measured mobility of the bilayer graphene was 2297 ± 3 cm^2^/Vs. This Cu NP-assisted graphene seeding growth technique enables high-quality Bernal-stacked bilayer graphene synthesis directly on insulating substrates, offering a promising pathway for advanced functional graphene device development.

## Data Availability

The original contributions presented in this study are included in the article/Supplementary Materials. Further inquiries can be directed to the corresponding authors.

## Supplementary Materials

The following supporting information can be downloaded at https://www.mdpi.com/article/10.3390/nano15211629/s1. Section S1: Catalytic Activity of Cu Nanoparticles (NPs); Figure S1: (a–c) SEM images of the commercial Cu NPs on carbon conductive adhesive. (d) Histogram of the particle size distribution based on statistical analysis of 200 NPs, together with a log-normal fit. The mean particle size is 23.7 nm, with a standard deviation of 7.9 nm; Figure S2: (a) Optical morphology of the as-transferred graphene sample on SiO_2_/Si substrate. (b) Optical morphology of the same region in (a) after graphene growth for 1h without NPs remote catalysis. (c) Optical morphology of the same region in (a) after graphene growth for 30 min with Cu NPs remote catalysis. The as-grown bilayer graphene domain is indicated by the arrow in (c); Figure S3: (a,b) SEM images of Cu NPs after graphene growth for 1 h and 3 h, respectively. (c) Raman spectra collected from the different areas of Cu NPs after graphene growth. (d) SEM image of a remote Cu foil after graphene grown for 1 h under the same growth conditions, where the Cu surface is completely covered by graphene. The inset shows the Raman spectrum of the as-grown graphene on the Cu foil; Section S2: First-principles DFT calculations; Figure S4: Atomic configurations of single carbon atom (a–d) and carbon dimer (e–h) adsorbed on monolayer and multilayer graphene; Section S3: Graphene growth on SiO_2_/Si; Figure S5: (a) SEM image of Cu nanoparticles (Cu NPs) after bilayer graphene growth under optimized CH_4_: H_2_ = 10:10 conditions, showing that part of the NP surfaces remain uncovered by graphene (Gr). (b) Raman spectra collected from the same region, where the red curve corresponds to graphene-covered areas and the black curve to uncovered surfaces, confirming incomplete graphene coverage and the persistence of catalytic activity; Section S4: Bilayer graphene growth on quartz and sapphire; Figure S6: SEM images of the bilayer graphene grown on monolayer graphene template-quartz (a) and sapphire substrates (b), respectively.
