# Supplementary material for: Direct Synthesis of Single-Crystalline Bilayer Graphene on Dielectric Substrate
Source: Nanomaterials (Basel). 2025 Oct 25;15(21):1629. doi: 10.3390/nano15211629 (PMC12608738; doi:10.3390/nano15211629)
Supplement: Supplementary file 1 [file nanomaterials-15-01629-s001.zip › nanomaterials-3902544-supplementary.pdf]

Supplementary Material

Direct synthesis of single-crystal bilayer graphene on dielectric substrate

Zuoquan Tan[1,2 #], Xianqin, Xing[1,2 #], Yimei Fang[3], Le Huang[4], Shunqing Wu[5], Zhiyong Zhang[4], Le Wang[1,2], Xiangping Chen[6, *] and Shanshan Chen[1,2 *]

[1] School of Physics and Beijing Key Laboratory of Optoelectronic Functional Materials and Micro-nano Devices, Renmin University of China, Beijing 100872, China

[2] Key Laboratory of Quantum State Construction and Manipulation (Ministry of Education), Renmin University of China, Beijing 100872, China

[3] School of Science, Jimei University, Xiamen 361021, China

[4] Key Laboratory for the Physics and Chemistry of Nanodevices and Center for Carbon-based Electronics, School of Electronics, Peking University, Beijing 100871, China.

[5] Collaborative Innovation Center for Optoelectronic Semiconductors and Efficient Devices, Department of Physics, Xiamen University, Xiamen 361005, China

[6] Tsinghua Shenzhen International Graduate School, Tsinghua University, Shenzhen, 518055, China

[#] Z. Tan and X. Xing contributed equally to this work.

*Correspondence: schen@ruc.edu.cn and cxp0826@sz.tsinghua.edu.cn

S1: Catalytic Activity of Cu Nanoparticles (NPs)

1.1 Morphology and size distribution of Cu NPs

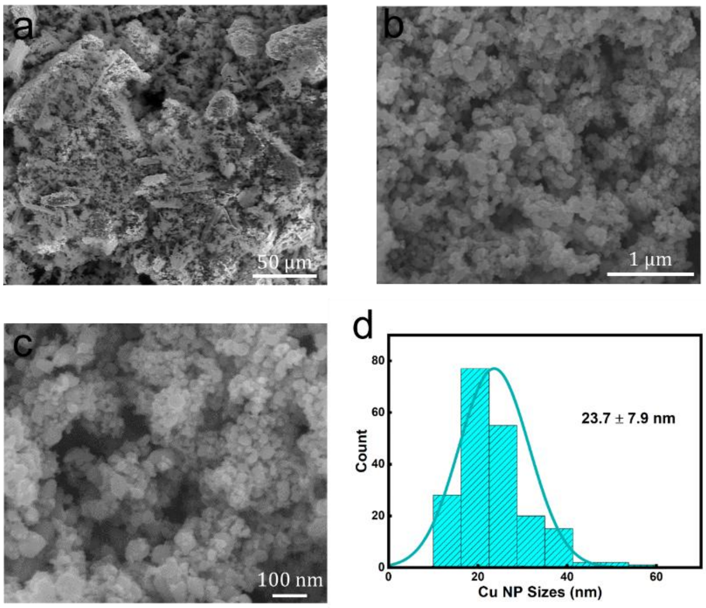

Figure S1. (a-c) SEM images of the commercial Cu NPs on carbon conductive adhesive. (d) Histogram of the particle size distribution based on statistical analysis of 200 NPs, together with a log-normal fit. The mean particle size is 23.7 nm, with a standard deviation of 7.9 nm.

1.2 Catalytic effect of Cu NPs

To isolate the role of the Cu nanoparticles, we ran a control experiment under otherwise identical conditions but with the Cu NPs removed (growth temperature 1000°C, CH_4:H_2 = 10:10). Figure S2a shows the as-transferred graphene film. Figure S2b shows the same region as in Figure S2a after 1h of graphene growth without the Cu NPs; no change is detectable on the surface. Repeating the 30-min growth with the Cu NPs introduced, we immediately observe a second graphene layer at the same locations, together with a high density of sub-micron dark spots that correspond to bilayer nuclei.

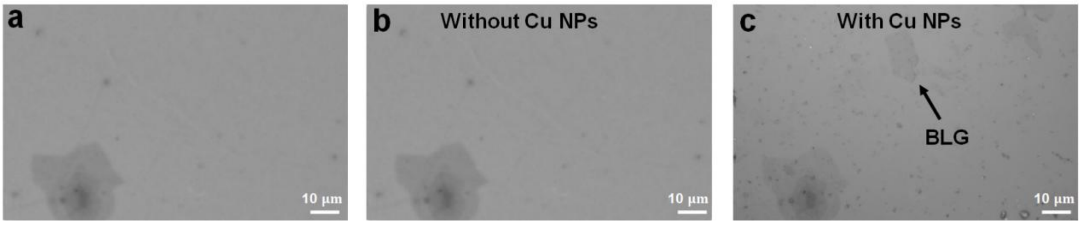

Figure S2. (a) Optical morphology of the as-transferred graphene sample on SiO_2/Si substrate. (b) Optical morphology of the same region in (a) after graphene growth for 1h without NPs remote catalysis. (c) Optical morphology of the same region in (a) after graphene growth for 30min with Cu NPs remote catalysis. The as-grown bilayer graphene domain is indicated by the arrow in (c).

1.3 Long-lasting catalytic activity of Cu NPs

To verify that Cu NPs remain catalytically active throughout an extended growth run, we carried out bilayer graphene synthesis under the optimized gas mixture (CH_4:H_2 = 10:10) for 1 h (a planar Cu foil was loaded alongside as a reference) and 3h, and subsequently examined the spent Cu NPs by SEM and Raman spectroscopy.

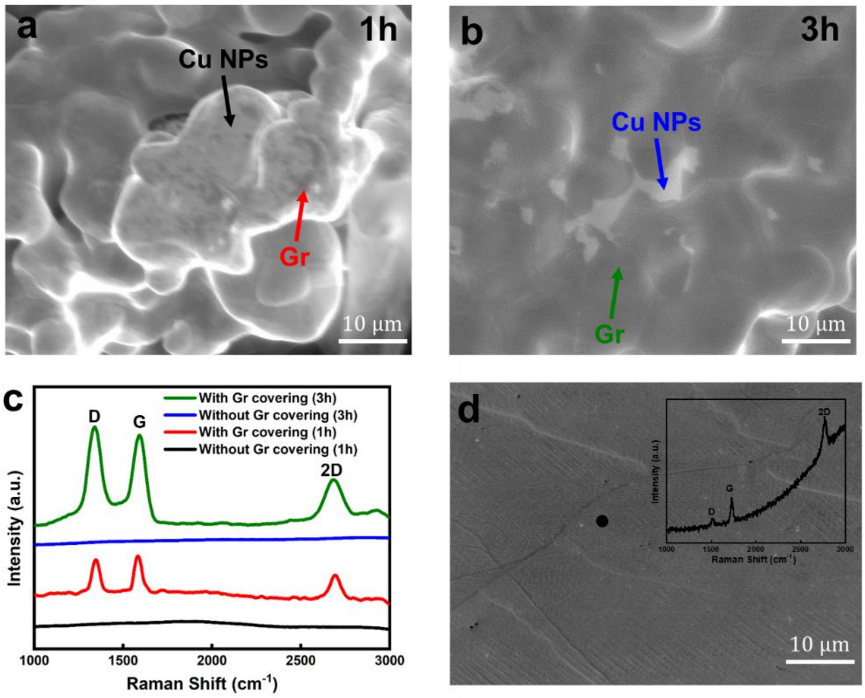

Figure S3. (a-b) SEM images of Cu NPs after graphene growth for 1 h and 3 h, respectively. (c) Raman spectra collected from the different areas of Cu NPs after graphene growth. (d) SEM image

of a remote Cu foil after graphene grown for 1 h under the same growth conditions, where the Cu surface is completely covered by graphene. The inset shows the Raman spectrum of the as-grown graphene on the Cu foil.

Figure S3 a, b show that the Cu-NP surface is only partially covered: darker patches correspond to graphene, whereas the brighter, bare Cu regions prove that catalytic Cu surface is still exposed after 1 h and 3h. Raman spectrum (Fig. S3c) corroborate this interpretation—pronounced G and 2D peaks are detected exclusively on the dark (covered) regions, while the bright (exposed) areas give featureless spectra. In contrast, the remote Cu foil is already fully coated with graphene after1 hour growth (Figure S3 d).

These results suggest that the Cu NPs provide continuous active catalysis during and even after extended growth, in sharp contrast to planar Cu foil where catalytic surface is rapidly encapsulated by graphene layer.

S2. First-principles DFT calculations

The adsorption energies of the first carbon atom adsorbed on multilayer graphene substrates (E_{ads}^{1st}) and the second atom adsorbed on multilayer graphene (E_{ads}^{2nd}) are defined as:

$$E_{ads}^{1st} = E_{total}^{one} - E_{multi} - E_{single} \,,$$

and

$$E_{ads}^{2nd} = E_{total}^{two} - E_{total}^{one} - E_{single} \,,$$

where E_{total}^{one} and E_{total}^{two} are total energies of one carbon atom and two carbon atoms adsorbed on multilayer graphene, respectively; E_{multi} is the energy of multilayers graphene, and E_{single} is the energy of single carbon atom.

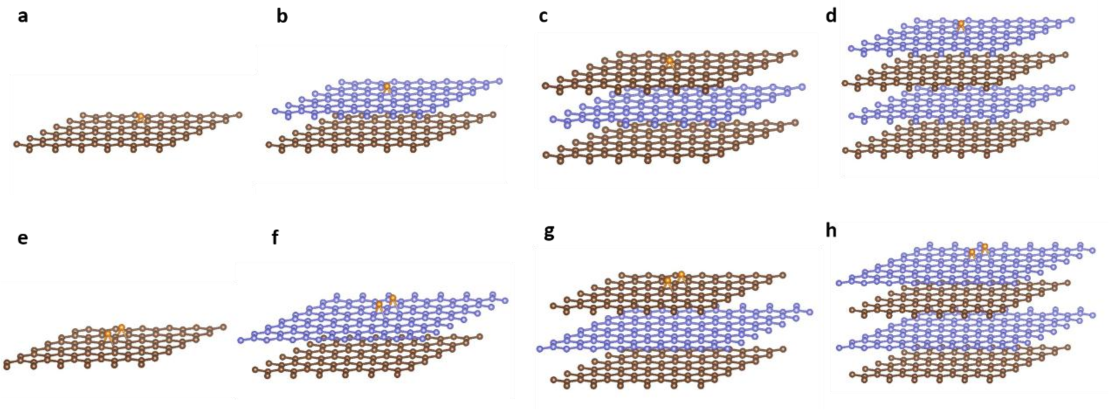

Figure S4. Atomic configurations of single carbon atom (a-d) and carbon dimer (e-h) adsorbed on monolayer and multilayer graphene.

S3: Graphene growth on SiO_2/Si

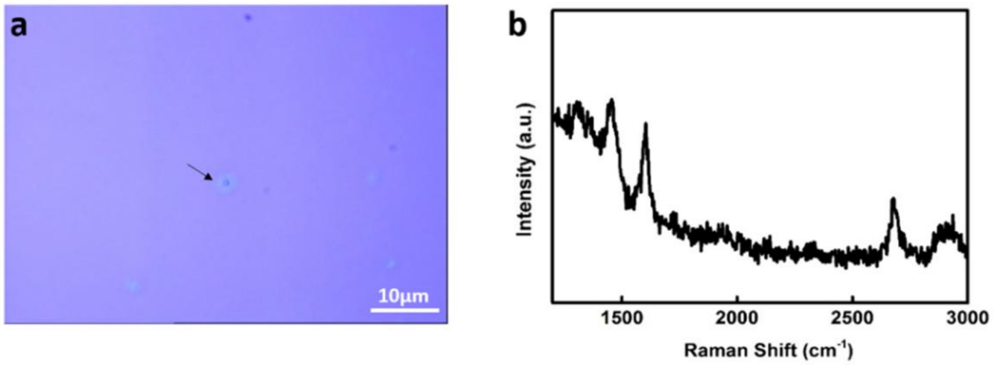

Figure S5. (a) SEM image of Cu nanoparticles (Cu NPs) after bilayer graphene growth under optimized CH_4:H_2 = 10:10 conditions, showing that part of the NP surfaces remain uncovered by graphene (Gr). (b) Raman spectra collected from the same region, where the red curve corresponds to graphene-covered areas and the black curve to uncovered surfaces, confirming incomplete graphene coverage and the persistence of catalytic activity.

S4: Bilayer graphene growth on quartz and sapphire

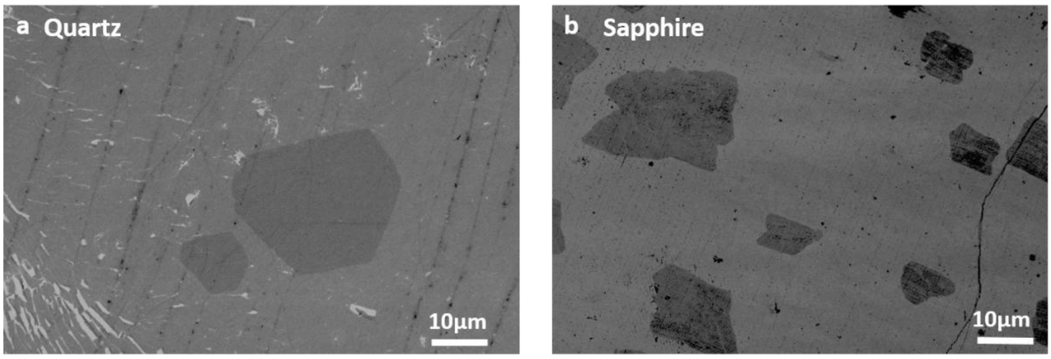

Figure S6. SEM images of the bilayer graphene grown on monolayer graphene template-quartz (a) and sapphire substrates (b), respectively.
